# Letter to the Editor: Cerebral CT perfusion dose: diagnostic reference levels should stimulate, not replace, optimization

**DOI:** 10.1186/s13244-026-02379-1

**Published:** 2026-09-08

**Authors:** Thomas Stein, Alexander Rau, Elias Kellner

**Affiliations:** 1 Departement of Diagnostic and Interventional Radiology, Medical Center, Faculty of Medicine, University of Freiburg, Freiburg, Germany; 2 Department of Neuroradiology, Medical Center, Faculty of Medicine, University of Freiburg, Freiburg, Germany; 3 Medical Physics, Department of Diagnostic and Interventional Radiology, Medical Center, Faculty of Medicine, University of Freiburg, Freiburg, Germany

Dear Editor,

We read the multicenter survey by Schegerer et al “International survey in four European countries on diagnostic reference levels (DRLs) based on clinical indications in computed tomography” with great interest and congratulate the authors on this timely and highly relevant contribution [1]. Their work addresses an important practical gap, as DRLs for many CT indications are either unavailable, insufficiently specific, or difficult to translate into daily protocol optimization. This is particularly relevant in stroke imaging, where CT protocols continue to show substantial interinstitutional variability.

The results for cerebral CT perfusion (CTP) are especially informative. Schegerer et al report a proposed CTP DRL of 190 mGy for CTDIvol and 2510 mGy⋅cm for DLP, with median values of 150 mGy and 1780 mGy⋅cm, respectively [1]. The authors state that these reported values remain below thresholds for deterministic effects, though remaining below such thresholds must not be equated with full protocol optimization. DRLs are primarily intended to identify unusually high practice and to stimulate review. They are therefore best understood as tools for optimization rather than as desirable target values.

Several findings reported by Schegerer et al make their study particularly useful in this regard. For CTP, the median intra-system dose variation was 1, mainly because most participating sites used fixed scan parameters [1]. This observation is important, because it suggests that dose levels are predominantly determined by local protocol design rather than by individualized patient adaptation. The authors also refer to an optimized approach in which sufficient temporal bolus coverage was achieved with CTDIvol values of 110–130 mGy, tube voltages of 70–80 kV, 24 rotations, and a total acquisition time of 45 s [1]. These values are below the proposed CTP DRL and below a considerable part of the routine practice captured in the survey. This indicates that additional optimization may be feasible in routine care, even when current practice remains formally DRL-compliant.

Previous dose-reduction work points in the same direction. Iterative reconstruction has enabled approximately half-dose cerebral CTP without relevant loss of objective or diagnostic image quality, although subjective image quality may be lower in some cases [2]. Low tube-current protocols around 50 mAs have produced perfusion measurements comparable to 100 mAs acquisitions in acute ischemic stroke, whereas 20 mAs appear less robust [3]. A recent single-center study also proposed local DRLs for multiphase CTA and CTP and reported that a local CTP protocol adjustment reduced dose while preserving diagnostic image quality [4]. In our own work, uniform reduction of temporal resolution to 3.0, 4.5, or 6.0 s reduced dose by factors of 2, 3, and 4, respectively, with only minor effects on perfusion metrics and relatively few changes in hypothetical thrombectomy decisions [5]. Taken together, these studies indicate that multiple technically realistic dose-reduction strategies for CTP are already available, although their degree of implementation in clinical routine remains heterogeneous.

The discussion by Schegerer et al on temporal sampling is also highly relevant. We agree that temporal sampling deserves particular attention as a potentially powerful lever for dose reduction. However, we would be cautious about fixed patient-independent non-uniform temporal sampling as a general solution. In a large cohort, we observed marked interindividual variation in arterial and penumbral bolus peak positions, with age-related delay but broad residual variance [6]. This has direct implications for protocol design. A fixed non-uniform sampling scheme assumes in advance when the diagnostically most relevant part of the bolus passage will occur. In acute stroke imaging, however, bolus timing is influenced by age, cardiac output, occlusion site, collateral status, and other patient-specific factors. More robust optimization pathways may therefore include uniform temporal downsampling, individualized bolus timing, reduced tube current or tube voltage where appropriate, and advanced reconstruction or denoising methods.

In this context, the comparatively high CTP dose values reported by Schegerer et al should not be seen as a limitation of the study, but rather as one of its important findings. The strength of the survey lies precisely in demonstrating that routine multicenter practice may remain substantially above levels that appear technically achievable under optimized conditions. The survey provides exactly the type of multicenter evidence needed to identify areas where routine practice may still differ from optimized practice. Accordingly, the proposed CTP DRL should be understood not as an endpoint of optimization, but as a trigger for it.

## Data Availability

Not applicable. No datasets were generated or analyzed for this article.
